# Recognition of medication information from discharge summaries using ensembles of classifiers

**DOI:** 10.1186/1472-6947-12-36

**Published:** 2012-05-07

**Authors:** Son Doan, Nigel Collier, Hua Xu, Pham Hoang Duy, Tu Minh Phuong

**Affiliations:** 1 National Institute of Informatics, Hitotsubashi, Chiyoda, Tokyo, Japan; 2 Department of Biomedical Informatics, School of Medicine, Vanderbilt University, Nashville, TN, USA; 3 Department of Computer Science, Posts and Telecommunications Institute of Technology, Hanoi, Vietnam

## Abstract

**Background:**

Extraction of clinical information such as medications or problems from clinical text is an important task of clinical natural language processing (NLP). Rule-based methods are often used in clinical NLP systems because they are easy to adapt and customize. Recently, supervised machine learning methods have proven to be effective in clinical NLP as well. However, combining different classifiers to further improve the performance of clinical entity recognition systems has not been investigated extensively. Combining classifiers into an ensemble classifier presents both challenges and opportunities to improve performance in such NLP tasks.

**Methods:**

We investigated ensemble classifiers that used different voting strategies to combine outputs from three individual classifiers: a rule-based system, a support vector machine (SVM) based system, and a conditional random field (CRF) based system. Three voting methods were proposed and evaluated using the annotated data sets from the 2009 i2b2 NLP challenge: simple majority, local SVM-based voting, and local CRF-based voting.

**Results:**

Evaluation on 268 manually annotated discharge summaries from the i2b2 challenge showed that the local CRF-based voting method achieved the best F-score of 90.84% (94.11% Precision, 87.81% Recall) for 10-fold cross-validation. We then compared our systems with the first-ranked system in the challenge by using the same training and test sets. Our system based on majority voting achieved a better F-score of 89.65% (93.91% Precision, 85.76% Recall) than the previously reported F-score of 89.19% (93.78% Precision, 85.03% Recall) by the first-ranked system in the challenge.

**Conclusions:**

Our experimental results using the 2009 i2b2 challenge datasets showed that ensemble classifiers that combine individual classifiers into a voting system could achieve better performance than a single classifier in recognizing medication information from clinical text. It suggests that simple strategies that can be easily implemented such as majority voting could have the potential to significantly improve clinical entity recognition.

## Background

Named Entity Recognition (NER) is an important step in natural language processing (NLP). It has many applications in the general language domain such as identifying person names, locations, and organizations. NER is crucial for biomedical literature mining as well [1,2]; many studies have focused on biomedical entities, such as gene/protein names. There are mainly two types of approaches to identifying biomedical entities: rule-based and supervised machine learning based approaches. While rule-based approaches use existing biomedical knowledge/resources, supervised machine learning based approaches rely heavily on annotated training data and domain dictionaries. The advantage of rule-based approaches is that they are easily customized to new vocabulary and author styles, while supervised machine learning approaches often report better results when the task domain does not change and training data is plentiful. Among supervised machine learning methods, Support Vector Machine (SVM) and Conditional Random Field (CRF) are the two most common algorithms that have been successfully used in NER in general and in the biomedical domain in particular [2-7].

One way to harness the advantages of both these approaches is to combine them into an ensemble classifier [4,6,8]. Zhou et al. [8] investigated the combination of three classifiers, including one SVM and two discriminative Hidden Markov Models, into an ensemble classifier with a simple majority voting strategy. They reported the best result for the protein/gene name recognition task in BioCreAtIvE task 1A (for gene mention identification) in comparison to other results. Smith et al. [4] showed that most of the top NER systems in the BioCreAtIvE II challenge for gene mention tagging combined results from multiple classifiers using simple heuristic rules. In a similar way, Torii et al. [6] used a majority voting scheme to combine recognition results from four systems into a single system called BioTagger-GM and reported a higher F-score than the first-place system in the BioCreAtIvE II challenge.

The 2009 i2b2 NLP challenge aimed to extract medication information (i.e., medication name, dosage, mode, frequency, duration, and reason) from de-identified hospital discharge summaries [9]. The different types of information were called *fields* and are described in Table 1. Note that fields might include phrases without noun phrases such as “as long as needed” or “until the symptom disappears”. The challenge asked that participating systems be used to extract the text corresponding to each of the fields for each medication mention. Among the top ten systems achieving the best performance in the 2009 i2b2 challenge, there were two machine learning based systems: the Sydney team ranked first while the Wisconsin team ranked tenth in the final evaluation [9]. Both systems used a similar volume of training data: 145 notes for the Sydney team and 147 notes for the Wisconsin team, respectively. The difference between those training data was that the Sydney team chose the 145 longest notes while the Wisconsin team randomly selected 147 notes from the training data [10]. The second best system, the Vanderbilt team, used a rule-based system which extended their MedEx system [11]. More recently, the i2b2 organizers used a maximum entropy (ME) model on the same training data as the Sydney team and reported that their results were comparable to the top systems in the challenge [12,13].

**Table 1:** Number of fields and descriptions with examples from the i2b2 2009 dataset

**Fields**	** #**	**Example**	**Description**
Medication	12773	“Lasix,” “Caltrate plus D,” “fluocinonide 0.5% cream,” “TYLENOL (ACETAMINOPHEN)”	Prescription substances, biological substances, over-the-counter drugs, excluding diet, allergy, lab/test, alcohol.
Dosage	4791	“1 TAB,” “One tablet,” “0.4 mg,” “0.5 m.g.,” “100 MG,” “100 mg x 2 tablets”	The amount of a single medication used in each administration.
Mode	3552	“Orally,” “Intravenous,” “Topical,” “Sublingual”	Describes the method for administering the medication.
Frequency	4342	“Prn,” “As needed,” “Three times a day as needed,” “As needed three times a day,” “x3 before meal,” “x3 a day after meal as needed”	Terms, phrases, or abbreviations that describe how often each dose of the medication should be taken.
Duration	597	“x10 days,” “10-day course,” “For ten days,” “For a month,” “During spring break,” “Until the symptom disappears,” “As long as needed”	Expressions that indicate for how long the medication is to be administered.
Reason	1534	“Dizziness,” “Dizzy,” “Fever,” “Diabetes,” “frequent PVCs,” “rare angina”	The medical reason for which the medication is stated to be given.

From the perspective of supervised machine learning, the medication extraction task in the 2009 i2b2 challenge can be divided into two steps: 1) identifying fields, and 2) determining the relationships between the detected medication names and the other fields. The first task was treated as a sequence labeling task and fields were considered as named entities [10,13]. In this paper, for the sake of convenience, we refer to the term “named entities” or “entities” as fields which have the same meaning as [10,13]. The first task was an NER task, for which both the Sydney and the Wisconsin teams used CRF to detect fields, while Halgrim et al. [13] used maximum entropy based classifiers. Using the test data in the challenge, Doan and Xu [14] investigated using output from the MedEx rule-based system as features for SVM algorithms and showed that those features could substantially improve an SVM-based NER system. However, the combination of multiple classifiers into an ensemble classifier presents another opportunity to improve NER performance on the i2b2 task, but it has not been investigated yet. To the best of our knowledge, this is the first study on investigating ensemble classifiers in recognizing medication relevant entities in clinical text.

In this study, we consider a fresh NER problem for clinical text in the 2009 i2b2 challenge and examine the combination of three classifiers: a rule-based system (MedEx, the second-ranked system in the 2009 i2b2 challenge), an SVM-based NER system, and a CRF-based NER system. Ensemble classifiers are built based on different combination methods and are evaluated using the challenge data set. Our studies provide valuable insights into the NER task for medical entities in clinical text. Throughout the paper, we compare our results against the top-ranked state-of-the-art system in the i2b2 challenge task from the Sydney group.

## Methods

### Data sets

**Figure 1:**
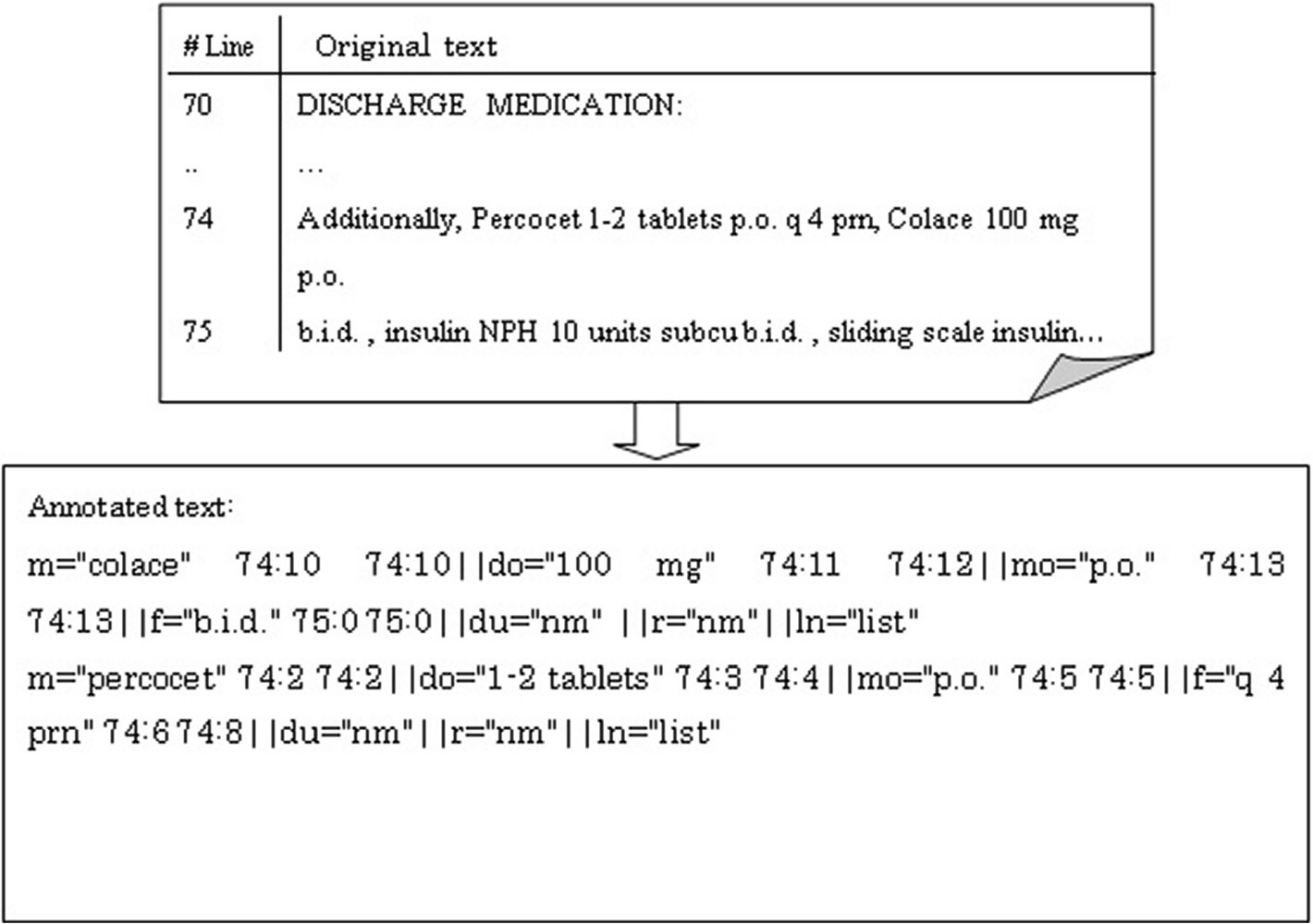
An example of the i2b2 data: “m” is for medication, “do” is for dosage, “mo” is for mode, “f” is for frequency, “du” is for duration, “r” is for reason, “ln” is for “list/narrative”.

At the beginning of the 2009 i2b2 challenge, a data set of 696 notes was provided by the organizers. In that set, 17 notes were annotated by the i2b2 organizers, based on annotation guidelines (see Table 1 for examples of medication information provided), and the rest were un-annotated. Participating teams developed their systems based on the training set, and they were allowed to annotate additional notes. 251 notes were randomly picked by the organizers as the test data set and were annotated by participating teams, as well as the organizers, and they served as the gold standard for evaluating the performance of systems [9,15]. An example of an original text and the annotation are shown in Figure 1.

### Rule-based method

MedEx was originally developed at Vanderbilt University for extracting medication information from clinical text [16]. It has two main components: a semantic tagger which uses a lexicon and rules to recognize entities, and a parser which uses a semantic grammar to output structured medication information. MedEx labels medication-related entities with a pre-defined semantic category, which overlaps with the six fields defined in the i2b2 challenge. The two semantic schemas are different but compatible. When the Vanderbilt team applied MedEx to the i2b2 challenge, they customized and extended MedEx to label medication-related fields as required by i2b2 [11]. There are three main customizations: 1) developing dedicated rules to combine entities recognized by MedEx into i2b2 fields, 2) adding a new section tagger and a spell checker, and 3) developing a post-processing program to convert MedEx outputs into the i2b2 challenge outputs.

The version of MedEx used in the 2009 i2b2 challenge used only 17 notes for customization. In this project, we used the 145 notes from the Sydney training data to customize the MedEx system. The changes were 1) adding new phrases for NEs from the Sydney training data into MedEx's dictionary, and 2) extending the post-processing program used in the 2009 i2b2 challenge. We refer to this rule-based method as “customized MedEx*.*”

### Machine learning-based methods

Named entity recognition is typically formulated as a sequence labeling problem which can be defined as follows: given a sequence of input tokens **x** *=* (*x*_1_*… x*_*n*_), and a set of labels *L*, determine a sequence of labels **y** *=* (*y*_1_*, …, y*_*n*_) for the tokens such that *y*_*i*_ ∈ *L* for 1 ≤ *i ≤ n*. In the case of named entity recognition, the labels incorporate two concepts: the type of the entity and the position of the token within the entity. In this project, we used a simple representation for token position called BIO. In BIO representation, a token (a word in our case) is labeled by one of three types of labels: **B** means the token is at the beginning of a field, **I** means the token is inside a field, and **O** means the token is outside any field [17]. Because we have six types of fields, we have six different **B** classes and six different **I** classes. For example, for medication names, we define the **B** class as “**B-m,**” and the **I** class as “**I-m.**” Therefore, we have 13 possible labels for each token (including the **O**). An example of the BIO representation is shown in Table 2. In the following section we describe the use of SVM and CRF for assigning a label to each token (word) from the discharge summaries.

**Table 2:** Statistics for the i2b2 2009 dataset

# Notes	268
# Sentences	9,689
# Words	326,474
# Fields	27,589

### Support vector machines

A Support Vector Machine (SVM) is a machine-learning method that is widely used in many NLP tasks such as phrase chunking, Part-of-Speech (POS) tagging, and NER. Essentially, it constructs a binary classifier using labeled training samples. Given a set of training samples, the SVM training phase tries to find the optimal hyperplane, which maximizes the distance of the training samples nearest to it (called support vectors). SVM takes as input a vector and maps it into a feature space using a kernel function [18,19].

In this paper we used TinySVM along with Yamcha, developed at NAIST [20,21]. We used a polynomial kernel function of degree 2, a *c*-value of 1.0, and a context window of +/−2. The *c*-value controls the tradeoff between errors of the SVM on training data and margin maximization. These values were the best parameters we obtained after testing with different polynomial kernels (*d* = 1,2,3,4,5) and *c*-values (from 0.5 to 5.0, at intervals of 0.5), using 10-fold cross-validation on all 268 annotated notes. 10-fold cross-validation was implemented as follows. All 268 discharge summaries were randomly divided into ten subsets of almost equal size. Nine subsets were used for training and the remaining subset was used for testing. We ran this procedure ten times; each time we picked a different subset for testing and the nine remaining subsets for training. Each time we built a classifier with the training sets and evaluated on the testing set. Reported results for SVM in this paper use the same parameter settings of SVM. We used the pairwise strategy for classification (one-against-one), i.e., the classifier will build *K*(*K*-1)/2 binary classifiers in which *K* is the number of classes (in this case *K* = 13). Each binary classifier will determine whether the sample should be classified as one of the two classes. Each binary classifier has one vote and the final output is the class with the maximum number of votes. These parameters have been used in many biomedical NER tasks such as [3,5,7].

### Conditional random field

Conditional Random Fields (CRFs) are probabilistic graphical models widely used for sequence labeling problems in general and NER in particular because they often achieve high performance [4,10,22]. A CRF assigns probabilities to possible class labels **y** of input sequences **x**. For the problem at hand, **x** is the sequence of tokens and **y** is the corresponding label sequence**.** In a linear chain CRF, which is used for sequence modeling tasks, the label *y*_*i*_ at position *i* depends on its predecessor *y*_*i*-1_ and successor *y*_*i*+1_.

The conditional probability of label sequence **y** given input sequence **x** is defined as

(1) $$P\left(y|x\right)=\frac{\exp(\sum_{i=1}^{L}\sum_{k=1}^{K}w_{k}f(y_{i-1},y_{i},i,x))}{\sum_{y^{'}}\exp(\sum_{i=1}^{L}\sum_{k=1}^{K}w_{k}f(y{'}_{i-1},y{'}_{i},i,x))}$$

in which the weights *w*_*k*_ are learned from training data, *L* is the length of the input sequence, *y*_*i*_ is the label at position *i*, *f*_*k*_ (.) is a feature function, and *K* is the number of feature functions. Once the weights are learned, one can label a new sequence by finding the most probable label sequence according to *P*(**y**|**x**). This decoding stage can be done in an efficient way using the Viterbi algorithm. In this study, we used the CRF++ tool which was developed at NAIST and is freely available [23]. CRF++ uses a combination of forward Viterbi and backward A* search. Below we describe the features of the CRF and SVM models.

### Feature sets

Different types of features for the SVM-based and CRF-based NER systems were investigated. For the SVM-based NER system, we used 1) words; 2) POS tags; 3) morphological features; 4) orthographies of words; 5) semantic tags determined by MedEx; and 6) history features. Details are described below:

•Word features: words only.

•POS features: Part-of-Speech tags of words. To obtain POS information, we used a generic POS tagger in the NLTK package [24].

•Morphologic features: suffix/prefix of up to 3 characters within a word.

•Orthographic features: information about whether a word contains capital letters, digits, special characters etc. We used the orthographic features described in [25] and modified some specifically for medication information such as “digit” and “percent.” In total, we had 21 labels for orthographic features.

•History features (or preceding class feature): class assignment of the preceding word.

•Semantic tag features: semantic categories of words. Typical NER systems use dictionary lookup methods to determine semantic categories of a word (e.g., gene names in a dictionary). In this study, we used MedEx, the best rule-based medication extraction system in the 2009 i2b2 challenge, to assign medication specific categories into words.

For the CRF-based NER system, we used 1) words; 2) POS tags; 3) morphological features; 4) orthographies of words; 5) semantic tags determined by MedEx; and 6) bigram features. We note that these features are different from those of the Sydney team when they used CRF-based methods. The Sydney team used six features corresponding to six NE classes and a window size of five. In order to extract those features, they used heuristic methods such as dictionary or gazetteer look-up for NE phrases [10]. In our opinion, their methods for extracting features are complicated. Their features are specific, which allowed them to achieve good performance in the i2b2 challenge, but they are not common features used for NER in the biomedical domain and may not be generalizable to other tasks.

### Combining classifiers' output in ensemble classifiers

In addition to using semantic tags provided by MedEx as input for SVM and CRF (which is equivalent to cascading the rule-based system with each of the two machine learning systems), we also explicitly combined the outputs of the three individual systems as an ensemble classifier. In previous work, various strategies have been proposed to integrate multiple classifiers, e.g. simple majority voting [6,8], bootstrapping [26] and boosting [27]. In this paper, ensembles were constructed using simple voting strategies. Below we describe voting strategies in detail.

#### Simple majority voting

The system assigns to each word the label that receives the most votes from the three individual systems (i.e. if at least two individual systems output the same label *y* for a given word *x*, *y* will be chosen as the final label for *x)*. If there is no agreement among the three systems, we assign the label predicted by the method which has the highest overall F-score for the current word. Simple and easy to implement, majority voting provides an effective way to combine classifiers and has been shown to deliver good performance in a number of applications, for example, in recognizing protein/gene names from research articles [8]. However, the effectiveness of this voting scheme in recognizing clinical entities has not been investigated, which we are addressing in this study.

Other ensembles were constructed based on characteristics of NE fields and our results of single CRF-based or SVM-based NER systems for individual fields in the first experimental setting (described below), especially for duration and reason. The six types of NEs are very different in terms of structured formats: mentions of medication, dose, frequency and mode often appear in structured forms as shown in Table 1, whereas duration and reason are often mentioned without noun phrases such as “as long as needed.” Therefore, it is difficult for one single machine learning model to perform well on all NE classes. Understanding the properties of SVM-based and CRF-based systems related to duration and reason, we constructed ensembles that benefit those two sets of NEs as described below.

#### Local CRF-based voting

In this model, duration and reason mentions are identified by the CRF, while the remaining mentions come from the SVM. Essentially, for a given word, if the CRF-based system predicts duration or reason then use its label for the word; otherwise, use the SVM-based prediction.

#### Local SVM-based voting

Duration and reason are those from the SVM model, whereas the remaining mentions are those from the CRF outputs. Specifically, for a given word, if the SVM predicts duration or reason then use its label for the word; otherwise, use the CRF prediction.

### Experimental settings

To evaluate the performance of the various methods and features we used two experimental settings. In the first setting, 268 annotated discharge summaries from the 2009 i2b2 challenge were used (17 from the training set and 251 from the test set). This annotated corpus contains 9,689 sentences, 326,474 words, and 27,589 entities. As mentioned in the Background section, the corpus includes six types of fields, i.e., medication, dosage, mode, frequency, duration, and reason. The number of fields and descriptions of the corpus are shown in Table 1 and statistics summarizing the corpus are shown in Table 3. Annotated notes were converted into a BIO representation and different types of feature sets were used in an SVM and a CRF classifier for NER as described in Methods. Feature sets were extracted from common lexical features as described in [3,5,7,25] and semantic features from MedEx. Evaluation for this setting was done using 10-fold cross-validation.

**Table 3:** An example of the BIO representation

				
**DISCHARGE**			**MEDICATION:**	
**O**		**O**		
Additionally,		Percocet	1-2	Tablets
**O**		**B-m**	**B-do**	**I-do**
p.o.	Q	4	prn,	
**B-mo**	**B-f**	**I-f**	**I-f**	

In the second setting, we compared our system with the system proposed by Patrick and Li [10], the top-ranked system in the 2009 i2b2 challenge. Specifically, we trained our system on the same training set consisting of 145 annotated notes as used by [10] and tested on the 251 notes provided as the standard test set of the 2009 i2b2 challenge.

### Significance tests

In order to compare performance between different systems, we used an approximate randomization approach for testing significance as described in [9]. For a pair of outputs *A* (with *j* entries) and *B* (with *k* entries) from two different systems, we computed micro-average F-scores from each output and noted the difference in performance (*f = A − B*). We then combined *A* and *B* to a superset *C*. Then we randomly drew *j* entries from *C* without resampling and created the pseudo set of entries *A*_*i*_; the remainder of *C* forms the pseudo set of entries *B*_*i*_. We then calculate $f_{i}=A_{i}-B_{i}$ and the number of times that *f*_*i*_*– f*≥0 , for all *i* and divided this count by *n* to calculate the p-value between *A* and *B*. We set *n* = 1000 as in Uzuner et al. [9].

## Results and discussion

We measured Precision, Recall, and F-score metrics using the standard CoNLL evaluation script (the Perl program conlleval.pl) [28]. Precision is the ratio between the number of NEs correctly identified by the system and the total number of NEs found by the system; Recall is the ratio between the number of NEs found by the system and the number of NEs in the gold standard; and F-score is the harmonic mean of Precision and Recall. For the whole system, we used micro-averaging of the Precision, Recall, and F-score. The micro-average is a weighted average over all NEs, where the weight for each NE is proportional to its size within the set of all NEs.

Experiments were run in a Linux machine with 4GB RAM and 4 cores of Intel Xeon 2.0GHz.

### Results for the first setting: 10-fold cross-validation

First, we evaluated the effectiveness of the features as well as their combinations. Table 4 shows Precision, Recall, and F-score of the SVM-based NER system for each type of entity when different combinations of feature sets were used. For all tables, “ALL” means the set of all named entities with values corresponding to micro-averaged Precision/Recall/F-score. Table 4 shows that the best F-score achieved was 90.54% when using all features for the SVM-based NER system. Semantic tag features from MedEx contributed greatly to the performance (89.47% F-score) compared to the remaining features, like history (83.81% F-score) or orthography (86.15% F-score). The difference between using and not using semantic tags was significant according to the approximate randomization test described above. Similar results (not shown) were observed for the CRF-based system.

**Table 4:** Performance of the SVM-based system for different feature combinations in 10-fold cross-validation

**Words**	**History**	**Morphology**	**POS**	**Orthographies**	**Semantics tag**	**Pre/Re/F-score**
*						87.09/77.05/81.76
*	*					90.34/78.17/83.81
*		*				91.81/80.74/85.91
*			*			89.92/78.54/83.84
*				*		91.62/81.32/86.15
*					*	92.38/86.73/**89.47**
*	*	*				91.72/81.08/86.06
*	*	*	*			91.81/81.06/86.10
*	*	*	*	*		91.78/81.29/86.22
*	*	*	*	*	*	93.75/87.55/**90.54**

* indicates the corresponding feature in the first row is used. Rows indicate the combination of features and their micro-averaged Precision(Pre)/Recall(Re)/F-score. The best F-score is underlined. F-scores that are significantly higher (p-values < 0.05) than all F-scores from rows above are shown in bold.

Second, we compared the performance of MedEx, CRF-based, and SVM-based NER systems (the CRF-based and SVM-based systems used all six types of features). The results for each separate field and overall results (micro-averaged scores) are given in Table 5 and the results of significance test for differences in performance between the methods are shown in Table 6. As shown, using all features, CRF and SVM give significantly higher F-scores (the differences are significant according to randomization tests) than the customized MedEx system which served as the baseline in this experiment. This can be explained because CRF and SVM can harness the advantages both from the rule-based system (i.e., features from MedEx) and from machine-learning algorithms. We also note that overall (column ALL), the performances of the CRF-based and SVM-based systems are comparable: CRF achieved a 90.48% F-score and SVM achieved a 90.54% F-score. When considering each field separately, significant differences in performance between the two systems have been observed for the dosage, frequency, and duration fields: the SVM-based system performed better for dosage and frequency while the CRF-based system performed better for duration. For the remaining three fields, the SVM-based system achieved higher F-scores for medication and mode, and the CRF-based system scored higher for reason; however, the differences were not statistically significant.

**Table 5:** Results from the customized MedEx system, CRF (all features), SVM (all features) systems, Simple Majority, Local CRF-based and SVM-based voting in 10-fold cross-validation: “m” stands for medication, “do” for dosage, “mo” for mode, “f” for frequency, “du” for duration, “r” for reason

		**ALL**	** m**	** do**	** mo**	** f**	** du**	** r**
**Customized MedEx**	Pre	89.57	90.33	95.01	96.26	92.09	51.19	62.10
	Re	84.01	89.10	82.88	86.95	88.50	58.82	47.93
	F-score	86.67	89.68	88.50	91.32	90.16	54.20	53.78
**CRF**	Pre	94.38	93.99	96.47	97.63	95.61	77.40	79.34
	Re	86.92	90.38	89.42	92.11	91.38	62.13	43.41
	F-score	90.48	92.13	92.79	94.77	93.42	68.64	55.74
**SVM**	Pre	93.75	93.84	95.40	97.13	95.68	70.42	74.46
	Re	87.55	90.76	91.46	93.27	92.69	48.21	44.50
	F-score	90.54	92.26	93.38	95.14	94.14	56.89	55.50
**Simple Majority Voting**	Pre	93.99	93.62	96.39	97.27	95.44	73.11	77.91
	Re	87.17	90.72	89.71	92.45	91.96	53.97	45.82
	F-score	90.43	92.12	92.91	94.78	93.63	61.65	57.37
**Local CRF-Based Voting**	Pre	94.11	93.86	95.43	97.16	95.65	70.58	85.81
	Re	87.81	90.79	91.49	93.27	92.64	65.76	40.87
	F-score	90.84	92.28	93.40	95.16	94.11	67.78	55.01
**Local SVM-Based Voting**	Pre	93.32	93.88	95.40	97.16	95.65	70.58	70.27
	Re	88.19	90.79	91.44	93.24	92.64	65.76	46.99
	F-score	90.67	92.30	93.37	95.14	94.11	67.78	56.08

**Table 6:** Statistical significance tests for differences in performance using approximate randomization in 10-fold cross-validation

	**CRF**	**SVM**	**Simple Majority Voting**	**Local CRF-Based Voting**	**Local SVM-Based Voting**
**Customized MedEx**	all, m, mo, do, f, du, r	all, m, mo, do, f, du	all, m, mo, do, f, du, r	all, m, mo, do, f, du	all, m, mo, do, f, du
**CRF**		do, f, du	du	all, do, du	do, f
**SVM**			du	du	du
**Simple Majority Voting**				all, do, du	du
**Local CRF-Based Voting**					NS

The entries in cells indicate that the two systems are significantly different in F-scores for the whole system (all), medication (m), dosage (do), mode (mo), frequency (f), duration (du), and reason (r). NS means “not significant different”. Significance is decided at p = 0.05.

Among the six NE fields, duration and reason proved to be the most difficult, with F-scores not exceeding 69% and 58% respectively by all experimented methods. The reasons that both duration and reason fields received lower scores than others might be: 1) the training data was smaller than for the other fields: there are only 957 duration fields and 1,534 reason fields, compared to 12,773 medication fields, and 2) the definitions of the frequency and duration fields might confuse the classifiers, e.g., “as needed” is defined as frequency but “as long as needed” is defined as duration.

Among all experimented methods, local CRF-based voting achieved the highest overall F-score (90.84%), followed by local SVM-based voting (90.67%). These scores were higher than those of the two single classifiers CRF and SVM. According to randomization tests, the local CRF-based voting system performed significantly better than the single CRF system for overall, dosage and was significantly less accurate than the single CRF system in recognizing duration. The significant difference in performance between local CRF-based voting and single SVM has been observed only for duration, for which the two systems achieved F-scores of 67.78% and 56.89% respectively.

### Results for the second setting: The 2009 i2b2 challenge with Sydney training data

In order to compare our results to the first-ranked system (the Sydney team from the 2009 i2b2 challenge), we used the same training dataset from their system and applied it to the standard test set. To evaluate the NER performance of the Sydney team’s system, we picked up three submissions from them in the challenge and chose the best one (submission 3). For the sake of comparison, the results from the Sydney team, the customized MedEx, the single classifiers based on SVMs and CRFs, and the three combination methods are shown in Table 7. We also present the results of statistical significance tests for differences in performance between the methods in Table 8.

**Table 7:** Results from the first ranked system (Sydney), customized MedEx system, CRF (all features), SVM (all features) systems, Simple Majority, Local CRF-based and SVM-based voting on the test set from the 2009 i2b2 challenge: “m” stands for medication, “do” for dosage, “mo” for mode, “f” for frequency, “du” for duration, “r” for reason

		**ALL**	** m**	** do**	** mo**	** f**	** du**	** r**
**Sydney**	Pre	93.78	93.51	94.78	96.45	96.59	69.71	76.04
	Re	85.03	88.31	88.91	91.28	91.25	40.93	38.83
	F-score	89.19	90.84	91.75	93.80	93.85	51.58	51.41
**Customized MedEx**	Pre	89.51	89.97	94.95	96.23	92.18	50.94	62.31
	Re	84.95	89.68	84.04	88.14	89.80	60.62	47.87
	F-score	87.17	89.83	89.16	92.01	90.97	55.36	54.14
**CRF**	Pre	94.11	93.71	95.92	97.26	95.60	71.88	77.52
	Re	84.89	89.19	87.37	90.19	90.73	38.86	40.97
	F-score	89.26	91.39	91.44	93.59	93.10	50.45	53.63
**SVM**	Pre	93.35	93.98	94.79	96.56	95.37	68.73	68.54
	Re	85.42	89.18	88.73	91.01	91.71	38.34	40.75
	F-score	89.21	91.51	91.66	93.71	93.50	49.22	51.12
**Simple Majority Voting**	Pre	93.91	93.62	95.86	97.23	95.58	72.73	75.90
	Re	85.76	90.19	87.62	90.44	91.20	44.21	43.67
	F-score	89.65	91.87	91.55	93.71	93.34	54.99	55.44
**Local CRF-Based Voting**	Pre	94.20	93.96	94.84	96.56	95.27	74.07	83.39
	Re	85.11	89.18	88.80	91.01	91.71	34.54	37.13
	F-score	89.42	91.51	91.72	93.71	93.46	47.11	51.38
**Local SVM-Based Voting**	Pre	93.03	94.06	94.77	96.56	95.37	66.94	65.83
	Re	85.76	89.18	88.71	90.98	91.66	42.66	44.52
	F-score	89.24	91.55	91.64	93.69	93.48	52.11	53.12

**Table 8:** Statistical significance tests for differences in performance using approximate randomization on the test set from the 2009 i2b2 challenge

	**Customized MedEx**	**CRF**	**SVM**	**Simple Majority Voting**	**Local CRF-Based Voting**	**Local SVM-Based Voting**
**Sydney**	all, m, mo, do, f	m	m	all, m, du, r	m,du	m
**Customized MedEx**		all, m, mo, do, f, du	all, m, mo, do, f, du, r	all, m, mo, do, f	all, m, mo, do, f, du	all, m, mo, do, f, du
**CRF**			NS	all, m, du	du	du
**SVM**				all, du, r	NS	NS
**Simple Majority Voting**					du, r	all, du, r
**Local CRF-Based Voting**						du

The entries in cells indicate that the two systems are significantly different in F-scores for the whole system (all), medication (m), dosage (do), mode (mo), frequency (f), duration (du), and reason (r). NS means “not significant different”. Significance is decided at p = 0.05.

As in the first experimental setting, customized MedEx was behind the machine learning and ensemble systems and received the lowest overall F-score of 87.17% (column “All” in table 7). Tables 7 and 8 show that when used individually, the SVM-based and CRF-based methods, with semantic tags from customized MedEx as one of input features, were comparable to the Sydney team’s system; specifically, the SVM-based and CRF-based systems and the system from the Sydney team achieved 89.21%, 89.26%, and 89.19% overall F-scores respectively. At the same time, the highest and second highest overall F-scores came from two ensemble methods: majority voting achieved an 89.65% F-score and local CRF-based achieved an 89.42% F-score. The score of majority voting was significantly higher than any single method including the method from the Sydney team, as determined by the statistical tests (Table 8). When considering each field separately, majority voting consistently outperformed the single methods in recognizing duration and reason. For medication – the most numerous field – majority voting also performed significantly better than the method from the Sydney team, customized MedEx, and the CRF-based system. The two single CRF-based, SVM-based systems as well as two local CRF-based and SVM-based voting systems are not significantly different, except for some variations in F-scores for the duration field.

The two experimental settings showed empirical evidence that the ensemble classifiers outperformed single classifiers. The best system for 10-fold cross validation is the CRF-based voting system and the best system for held-out testing set is the majority voting. Since the training and testing data in the two experimental settings are different, it is difficult to choose the best voting method for all data.

## Conclusions

In this study, we investigated the combination of NER outputs from a rule-based system and two supervised machine learning systems into an ensemble classifier using different voting methods to improve recognition of medication entities from discharge summaries. Our experimental results using the 2009 i2b2 challenge datasets showed that ensemble classifiers that combine individual classifiers into a voting system could achieve better performance than a single classifier in recognizing medication related entities from clinical text. It suggests that simple strategies that can be easily implemented, such as majority voting, could have the potential to significantly improve clinical entity recognition. Further combinations of classifiers will be investigated in the future.

## Abbreviations

SVM: Support vector machine; CRF: Conditional random field; NER: Named entity recognition; POS: Part-of-speech; NLP: Natural language processing.

## Competing interests

The authors declare that they have no competing interests.

## Authors' contributions

SD, XH, and TMP developed the idea for this study. The project was supervised by XH, TMP, and NC. SD designed and carried out the experiments. Data analysis was conducted by SD, PHD, and TMP. The manuscript was prepared by SD with additional contributions by all authors. All authors read and approved the final manuscript.

## Pre-publication history

The pre-publication history for this paper can be accessed here:

http://www.biomedcentral.com/1472-6947/12/36/prepub
